# Direct synthesis of vertically aligned ZnO nanowires on FTO substrates using a CVD method and the improvement of photovoltaic performance

**DOI:** 10.1186/1556-276X-7-293

**Published:** 2012-06-06

**Authors:** Liyou Lu, Jiajun Chen, Lijuan Li, Wenyong Wang

**Affiliations:** 1 Department of Physics and Astronomy, University of Wyoming, P.O. Box 3905, Laramie, WY, 82071, USA

**Keywords:** nanowires, ZnO, direct growth, FTO-coated glass substrate, dye-sensitized solar cell

## Abstract

In this work, we report a direct synthesis of vertically aligned ZnO nanowires on fluorine-doped tin oxide-coated substrates using the chemical vapor deposition (CVD) method. ZnO nanowires with a length of more than 30 μm were synthesized, and dye-sensitized solar cells (DSSCs) based on the as-grown nanowires were fabricated, which showed improvement of the device performance compared to those fabricated using transferred ZnO nanowires. Dependence of the cell performance on nanowire length and annealing temperature was also examined. This synthesis method provided a straightforward, one-step CVD process to grow relatively long ZnO nanowires and avoided subsequent nanowire transfer process, which simplified DSSC fabrication and improved cell performance.

## Background

Dye-sensitized solar cells (DSSCs) have attracted significant research interest due to their promising power conversion efficiency and low fabrication cost [1]. Typical photoanodes of DSSCs are layers of nanoparticles of wide band gap semiconductors such as TiO_2_ or ZnO, and the substrates are usually fluorine-doped tin oxide (FTO)-coated glasses [2]. However, in these nanoparticle-DSSCs, photo-generated electrons have to percolate through the nanoparticle network before they reach the collection electrode, which increases charge recombination possibility and limits cell performance. One approach to improving charge collection efficiency in DSSCs is to replace the nanoparticle network with one-dimensional structures such as semiconductor nanowires that can provide direct transport pathway for the carriers. Due to the enhanced diffusion length, longer wires and thus thicker photoanode films can be incorporated into DSSCs, which could lead to better quantum efficiency in the long-wavelength region of the solar spectrum [3]. In addition, recent studies also show that the open-circuit voltage of DSSCs can be improved by employing nanowire-photoanodes, which is attributed to a suppressed back electron transfer reaction that occurs at the photoanode/redox electrolyte solution interface, highlighting the importance of exploring nanowire-based photoanodes for DSSC applications [4,5].

There has been a significant amount of reports on DSSCs based on nanowires, where the semiconductor nanowires are mainly synthesized using solution-based hydrothermal method [6-8]. Using this method, the nanowires can be directly grown on FTO-coated substrates, which make subsequent solar cell fabrication straightforward. However, solution-based synthesis is usually slow and involves multiple processes, and post-growth annealing is necessary to remove the unwanted chemicals from the nanowire surface and ensure good electrical contact between the wires and the substrate [9]. Furthermore, it is generally difficult to produce long wires using the hydrothermal approach. Due to the multiple steps involved and the low growth rate, it is very time consuming to synthesize nanowires with a length of more than 10 μm [10-12]. Another popular nanowire synthesis approach is the chemical vapor deposition (CVD) method that is based on vapor–liquid–solid (VLS) growth mechanism. Nanowires with very long length can be synthesized this way; however, the substrates used are typically silicon or sapphire other than FTO-coated glasses since the high CVD growth temperature can easily damage the transparent conducting oxide [13-15]. In addition, since the nanowires are not directly synthesized on FTO-coated substrates, a nanowire transfer process is needed in the subsequent solar cell fabrication. Such a transfer process causes contact issues between the nanowires and the substrate as well as broken wires in the device structure that creates additional transport barriers and recombination possibilities for photo-generated electrons, which all could limit solar cell performance.

Thus far, there is only limited research on direct synthesis of nanowires on FTO-coated substrates using the CVD method, and the reported nanowires were not aimed at DSSC applications and had low density and random morphology [16,17]. In this work, we investigated a controlled CVD synthesis of nanowires directly on FTO-coated glass substrates. Long, vertically aligned ZnO nanowires were fabricated at a relatively low temperature of 550 °C, and they formed dense arrays with length of tens of microns in a one-step vapor deposition process. DSSCs were fabricated using these directly grown nanowires, and the performance was compared to those fabricated using transferred ZnO nanowires. The effects of nanowire length and annealing temperature on device performance were also examined.

## Methods

### Direct growth of ZnO nanowires on FTO substrates by the CVD method

**Figure 1:**
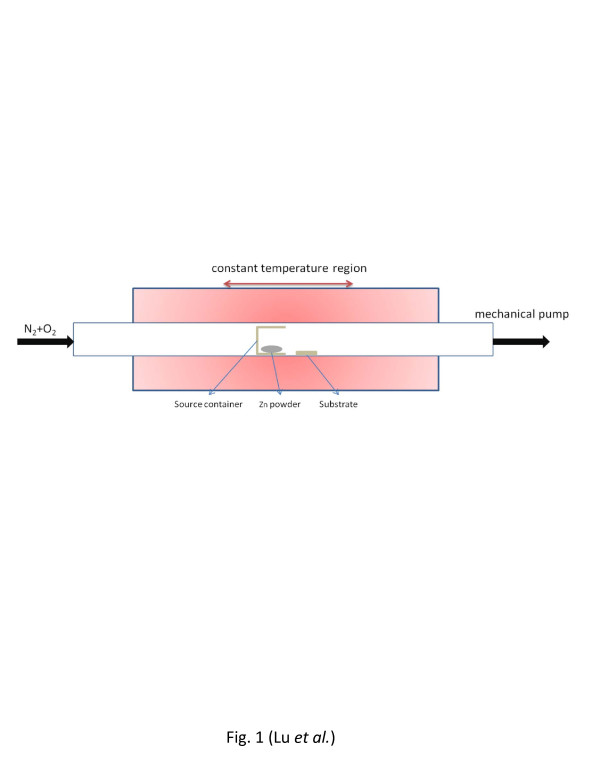
**Schematic of the system setup for nanowire synthesis.** (not drawn to scale).

Vertically aligned single crystalline ZnO nanowires were synthesized directly on FTO-coated glass substrates in a horizontal tube furnace at a low temperature by the chemical vapor deposition method, where the nanowire growth followed a self-catalytic vapor–liquid–solid mechanism [16,18,19]. Figure 1 shows the schematic of the system setup. A 1-in. quartz tube was mounted on a single-zone furnace with a constant temperature heating zone of about 13 cm long. As shown in Figure 1, a specially designed cylindrical sapphire source container with an inner diameter of 1.4 cm, outer diameter of 2.0 cm, and length of 2.5 cm was used in this experiment. The container with 0.3 g zinc powder (100 mesh, 99.9%, Alfa Aesar, Ward Hill, MA, USA) as the source material was placed at the center of the tube. FTO-coated glass substrate (TEC15, MTI, Richmond, CA, USA) with a size of 1.0 × 1.5 cm was first cleaned by acetone and isopropyl alcohol and then covered by a Si_3_N_4_ shadow mask with a 0.5 × 0.5 cm square opening at the center. The substrate, together with the shadow mask, was placed inside the tube at a distance of 0.5 cm downstream from the source container. The tube furnace was first pumped down to a base pressure of 10^−2^ Torr using a rotary pump, and then, it was heated up to 550 °C under a ramp rate of 50 °C/min and a carrying gas mixture of N_2_ (100 sccm) and O_2_ (4 sccm). The temperature was maintained at 550 °C, while the pressure was kept at 8 Torr to allow the nanowires to grow.

### Solar cell fabrication using ZnO nanowires directly synthesized on FTO substrates

The as-grown ZnO nanowires were ready for solar cell fabrication without any further processing. To sensitize the nanowires, the FTO substrate with the ZnO nanowires was soaked in a 0.05-mM solution of N719 dye (dissolved in dry ethanol; SOLARONIX, Aubonne, Switzerland) at 50 °C for 2 h. Another FTO substrate coated with 25 nm Pt was used as the counter electrode and was bonded together with the nanowire/FTO substrate through a hot-melt spacer (75 μm; Bynel, Dupont, Wilmington, DE, USA). A drop of electrolyte (0.5 M LiI (Aldrich, St. Louis, MO, USA), 50 mM I_2_ (Alfa Aesar), and 0.5 M 4-tertbutylpyridine (Aldrich) in 3-methoxypropionitrile (Aldrich)) was injected into the space between the two electrodes of the cell. Current density-voltage (*J*-*V*) curves were acquired by a source measurement unit (Agilent 4156 Semiconductor Parameter Analyzer, Agilent Technologies, Santa Clara, CA, USA) under a simulated sunlight (100 mW/cm^2^, calibrated by a KG-5 filtered silicon photodiode) using a setup with a Xenon lamp. The optical absorption of the dye solution was characterized by an ultraviolet–visible (UV–vis) spectrophotometer (Lamda 950, PerkinElmer, Waltham, MA, USA).

### Solar cell fabrication using ZnO nanowires transferred onto FTO substrates

DSSCs based on transferred ZnO nanowires were also fabricated and tested in this research for the purpose of a comparison study. Since it was very difficult to remove the directly synthesized nanowires from the FTO substrates, the transferred ZnO nanowires were those grown on silicon substrates. The solar cell fabrication procedure was almost identical, except that a nanowire transfer process was involved. To transfer the ZnO nanowires, a polydimethylsiloxane (PDMS) solution was first spin-coated on the silicon substrate with the ZnO nanowires, which, after annealing, would form a flexible but solid film that holds the nanowires in position [20,21]. After being annealed at 150 °C on a hot plate in the air, the nanowire film was peeled off by a sharp razor blade. The nanowire film was then soaked in the N719 dye solution for 2 h at 50 °C, which was the same sensitization condition for the DSSCs based on directly grown ZnO nanowires. After dye sensitization, the film was transferred onto an FTO substrate and was glued down using a thin layer of silver paste. Since the silver paste was easy to dissolve in a dye solution, dye sensitization was performed before the nanowire film attachment, which was different from the previously reported procedure [20].

## Results and discussion

### Direct synthesis of vertically aligned ZnO nanowires on FTO substrates by the CVD method

**Figure 2:**
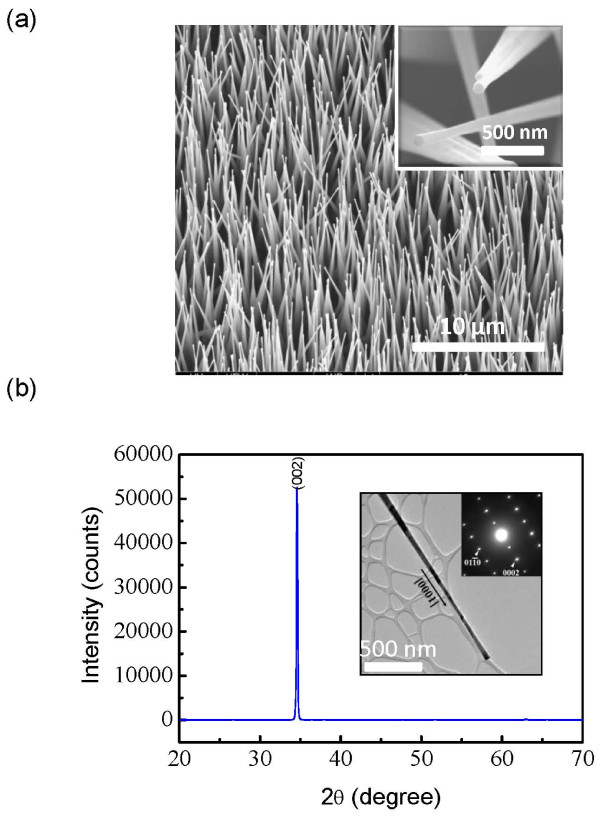
**Vertically aligned ZnO nanowires on FTO substrates.** (**a**) FESEM image (tilted at 15°) of a directly synthesized ZnO nanowire array on a FTO substrate. The inset is a higher-magnification image. (**b**) XRD pattern of the ZnO nanowires grown on the FTO substrate. The inset shows the transmission electron microscopy image of a single wire and the corresponding selected area electron diffraction pattern.

Figure 2a shows the field emission scanning electron microscope (FESEM) image (tilted at 15°) of the as-grown ZnO nanowires on an FTO substrate, and the inset is a higher-magnification image. The needle-shaped nanowires were vertically aligned, with a hexagonal face on the tip of each nanowire. The X-ray diffraction (XRD) pattern in Figure 2b reveals the single crystalline structure of the wires with a [0001] growth direction, which is consistent with the transmission electron microscopy examination of a single nanowire that is shown in the inset of Figure 2b. The top and bottom diameters of the needle-shaped ZnO nanowires were around 100 nm and 1 μm, respectively. The lengths of the nanowires were tens of microns, which could be controlled by adjusting the growth time. In our experiment, the nanowire growth was different from that of the conventional VLS process, where a catalyst (e.g., Au) is necessary to promote a uni-axial growth, and the lattice match between the nanowires and the substrate is critical for achieving vertically aligned nanowire arrays [22-24]. For the synthesis reported here, metal catalyst was not used and the growth followed a vapor-phase transport deposition process. The zinc powder first evaporated slowly after the furnace temperature was increased and formed a uniform thin seed layer of ZnO on the FTO substrate. The zinc vapor pressure became higher as the temperature was further increased, and a relatively high-zinc-concentration environment was formed around the substrate location, and the nanowire growth was initiated. It is important to point out that the source container played a critical role in the growth process. Since the size of the source container was only several millimeters smaller than the inner diameter of the tube, the container blocked the direct flow of the carrying gas over the zinc power and prevented the evaporated zinc vapor from being transferred too fast, which helped maintain the supersaturation level of the vapor that assisted the nanowire growth. In fact, when the diameter of the source container was modified to be smaller than the aforementioned dimension, the nanowire growth was significantly affected, and for certain cases, there was no nanowire growth at all. The synthesized nanowires showed very good mechanical attachment to the FTO substrates, and it was very difficult to remove the nanowires by the typical ultrasonic method.

**Figure 3:**
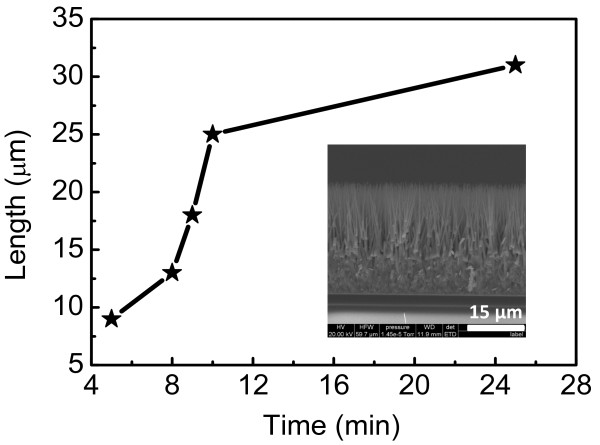
**ZnO nanowire array length dependence on the growth time.** Inset is a cross-sectional FESEM image of a nanowire array with a length of 31 μm.

ZnO nanowires with different lengths were synthesized in this research. The length control of the nanowire arrays was realized by adjusting the growth time only while keeping all the other growth parameters constant. Figure 3 exhibits the dependence of the nanowire array length on the growth time. The longest nanowire array obtained for this experiment was 31 μm under a growth time of 25 min. If the growth time was further increased, then more source material was needed in order to produce longer wires. The inset in Figure 3 shows a cross-sectional FESEM image of an as-grown ZnO nanowire array with a length of 31 μm on an FTO substrate.

### DSSCs based on directly synthesized and transferred ZnO nanowires

**Figure 4:**
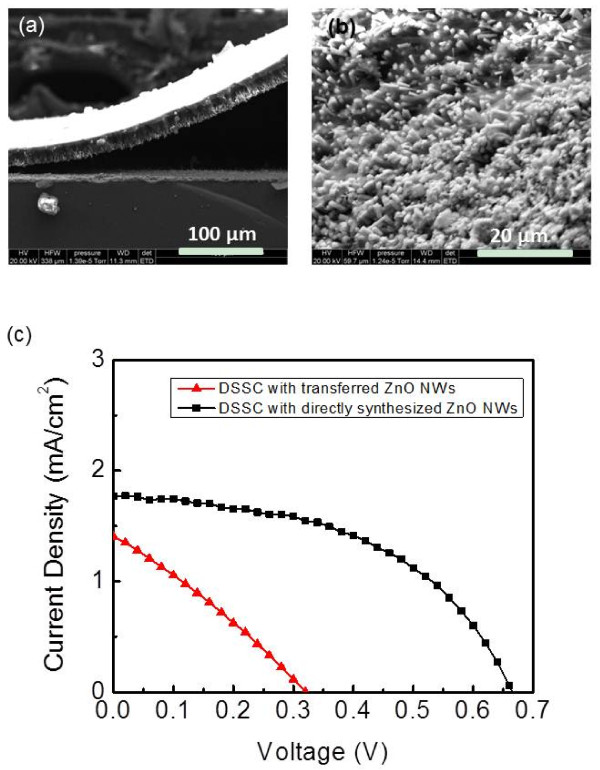
**DSSCs based on directly synthesized and transferred ZnO nanowires.** (**a**) Cross-sectional FESEM image of a ZnO nanowire film during the peeling-off process. (**b**) FESEM image of the bottom of the ZnO nanowire film after removing from the silicon substrate. (**c**) *J*-*V* plots of DSSCs fabricated using transferred and directly grown ZnO nanowires.

DSSCs were fabricated using both directly synthesized and transferred ZnO nanowires in order to compare their performance. Figure 4a is the cross-sectional FESEM image of a ZnO nanowire film during the removing process, where the major part of the film was peeled off but a small portion was still attached to the silicon substrate. Figure 4b shows the bottom of the ZnO nanowire film after it was removed from the Si substrate, which would be attached onto a FTO-coated substrate using silver paste. The advantages of this transfer procedure were the following: the whole nanowire array could be transferred at one time, the nanowires kept good vertical alignment [20], and the procedure did not cause a significant amount of broken wires inside the nanowire array. A typical *JV* curve of a DSSC fabricated using the transferred ZnO nanowires is shown in Figure 4c. After subtracting the thickness of the PDMS layer, the effective nanowire length for dye molecule loading of this device was 12 μm. The short-circuit current density (*J*_sc_) was 1.4 mA/cm^2^, and the open-circuit voltage (*V*_oc_) was about 0.32 V. These values were comparable to the previous reported results of DSSCs fabricated using the same nanowire transfer procedure [20]. As a comparison, Figure 4c also shows the *JV* curve of a DSSC fabricated using directly synthesized ZnO nanowires that had the same effective nanowire length of 12 μm for dye loading, which exhibited substantial improvement on both *J*_sc_ and *V*_oc_. One indicator of solar cell performance is the series resistance (*R*_s_) that can be estimated from a *JV* curve using $R_{\text{s}}=\frac{dV}{dJ}{|}_{J=0}$[25]. To enhance the output efficiency of a solar cell, the cell's series resistance should be minimized. The calculated *R*_s_ of the DSSC with transferred ZnO nanowires was 195 Ω·cm^2^, which was significantly larger than that of the cell fabricated using directly synthesized nanowires (85 Ω·cm^2^). The major contributions to the series resistance of nanowire-DSSCs are the resistance of the nanowire array, the contact resistance between the nanowires and the bottom FTO electrode, the resistance between the nanowires and the electrolyte, the resistances of the electrolyte and between the electrolyte and the counter FTO electrode, the resistances of the two FTO electrodes, and the parasitic probe resistance. The major difference in the device structures of the two types of DSSCs was the bottom contact between the nanowire array and the FTO-coated substrate, and this increase in the series resistance could be mainly attributed to the less-than-ideal bottom contact in the DSSCs fabricated using transferred nanowires.

### DSSCs based on directly synthesized ZnO nanowires with different lengths

**Figure 5:**
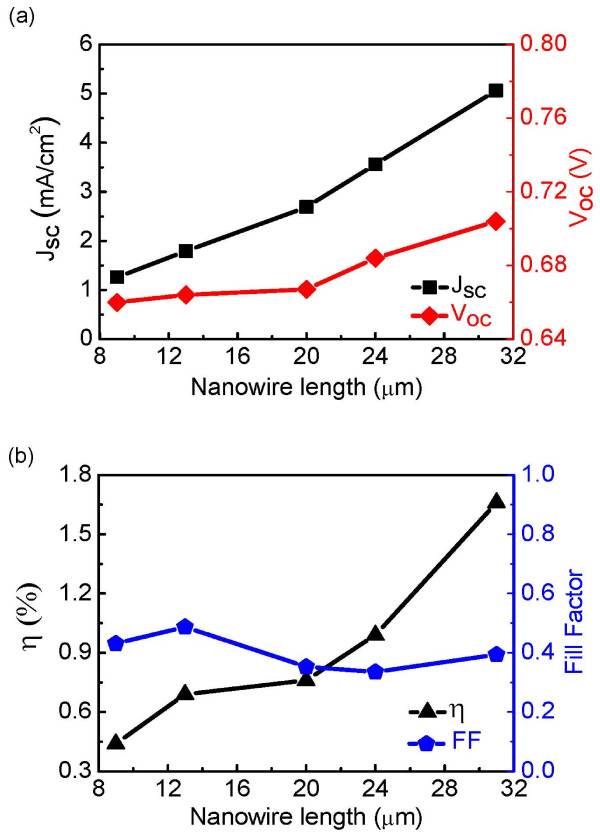
**Effect of the nanowire array length on cell performance parameters.** (**a**) Plots of short-circuit current density and open-circuit voltage as functions of nanowire array length. (**b**) The overall power conversation efficiency and fill factor as functions of nanowire array length.

DSSCs have been fabricated using the directly synthesized ZnO nanowires with different lengths. Figure 5 shows the effect of the nanowire array length on cell performance parameters including *J*_sc_*V*_oc_, power conversion efficiency (*η*), and fill factor (FF). As Figure 5a,b shows, *J*_sc,_*V*_oc_, and *η* of the DSSCs were improved when the nanowire array length became longer, which could be explained by the increase in dye molecule loading due to the longer wires used. The cell fabricated with the longest nanowires showed the best performance of *J*_sc_ of 5.1 mA/cm^2^*V*_oc_ of 0.71 V, and *η* of 1.7%. This performance was among the best of ZnO nanowire-DSSCs [7,9,26,27].

### Effect of annealing on the performance of DSSCs

**Figure 6:**
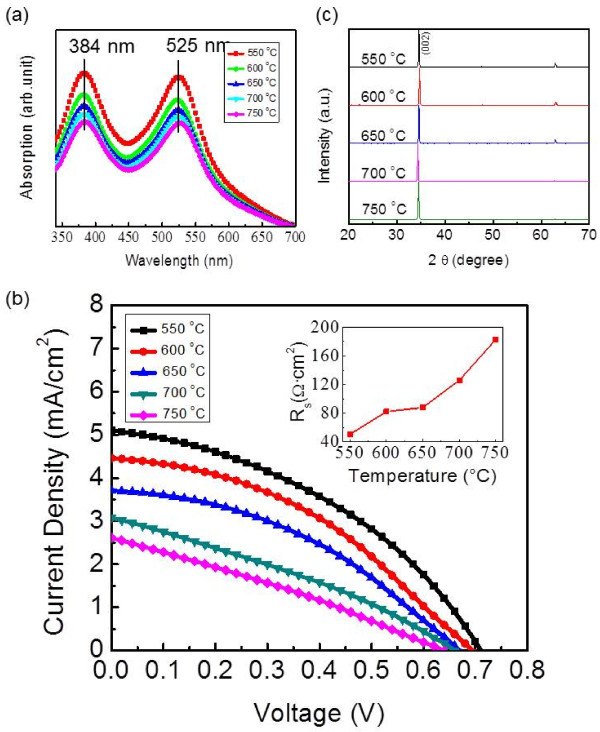
**Annealing effects on the performance of DSSCs.** (**a**) UV–vis absorption spectra of the N719 dye solutions after nanowire sensitization. The temperature represents the nanowire annealing temperature. (**b**) *J*-*V* curves of the DSSCs fabricated using as-grown nanowires on FTO substrates annealed at different temperatures. The inset shows the dependence of the series resistance on the annealing temperature.(**c**) XRD data of ZnO nanowires on FTO substrates annealed at different temperatures.

To investigate possible methods to enhance solar cell performance, the effect of annealing on device performance was also studied. Directly synthesized ZnO nanowires on FTO substrates with the same length of 31 μm were annealed at 550, 600, 650, 700, and 750 °C, respectively, under the same growth-forming gas environment, and DSSCs based on the annealed nanowires were fabricated. To examine the annealing effect on dye molecule loading, the absorption spectra of the N719 dye solutions after nanowire sensitization were measured by a UV–vis spectrophotometer, and the results are shown in Figure 6a. The temperatures in Figure 6a represent the different nanowire annealing temperatures, and the two peaks at 384 and 525 nm are the characteristic absorption peaks of the N719 dye. A higher absorption intensity in Figure 6a corresponded to a larger amount of dye molecules left in the solution after nanowire sensitization, thus indicating a smaller amount of dye loading on the nanowire surface. As Figure 6a reveals, when the annealing temperature was increased, there was more dye molecule loading on the ZnO nanowires. However, despite of the improved dye loading at higher annealing temperatures, the fabricated solar cells actually showed decreased performance, as Figure 6b shows. The inset in Figure 6b shows the calculated series resistances of the cells with annealed nanowires. As it reveals, *R*_s_ increased significantly when the annealing temperature was increased. Figure 6c shows the XRD data of ZnO nanowires on FTO substrates annealed at different temperatures, suggesting that the crystalline structure of the ZnO nanowires did not change significantly after annealing. The increase in the series resistance was possibly due to the high-temperature annealing damage to the FTO-coated substrates. We carried out a control study of bare FTO-coated substrates annealed at same temperatures using the typical 4-point probe measurement, and the FTO substrates' surface resistivities showed an increase at high annealing temperatures. However, this increase alone could not justify the significant change in the cell's series resistance. Another possibility could be an increased formation of Zn^2+^ dye clusters on the nanowire surface after high-temperature annealing [28]. Such clusters could create additional barriers for electron transfer from the dye molecules to the nanowires and cause degradation in device performance [28-30].

## Conclusions

In this research, we demonstrated a method to directly synthesize vertically aligned long ZnO nanowires on FTO-coated glass substrates. The synthesis is based on a straightforward, one-step CVD approach, which avoided the wet chemical processing in typical hydrothermal growth and eliminated the nanowire transfer process for DSSC fabrication. DSSCs based on these directly grown ZnO nanowires showed improved performance compared to those fabricated using transferred nanowires. The performance of the DSSCs could be further improved when longer nanowires were used. The relatively long nanowires provided an alternative for hybrid nanowire/composite solar cells in efficiency enhancement [31,32]. The effect of the annealing temperature was also examined, and it was observed that high annealing temperature caused a substantial increase in the cell's series resistance and lowered the device performance. The reported direct synthesis approach could be further improved and applied for the growth of other types of nanowires and could benefit the fabrication of dye- or quantum dot-sensitized solar structures.

## Competing interests

The authors declare that they have no competing interests.

## Authors' contributions

LL performed the experiment and drafted the manuscript. JC and LL participated in the experiment. WW supervised the work and finalized the manuscript. All authors read and approved the final manuscript.
